# Natuurlijk naar buiten!

**DOI:** 10.1007/s12508-021-00310-1

**Published:** 2021-07-12

**Authors:** Vasanthi Iyer, Clair Enthoven, Caroline Klaver, Edith Mulder, André Soeterbroek, leden van ‘Zicht op Buiten’

**Affiliations:** 1 TNO, Leiden, Nederland; 2 afdeling Oogheelkunde, Erasmus MC, Rotterdam, Nederland; 3 afdeling Oogheelkunde, Radboudumc, Nijmegen, Nederland; 4 Diabetesvereniging Nederland, Leusden, Nederland; 5 Houding Netwerk Nederland, Nijkerk, Nederland

**Keywords:** leefstijl, buitenshuis, kinderen, gezondheid, preventie, Lifestyle, Outdoors, Children, Health, Prevention

## Abstract

Vaker buiten spelen maakt kinderen gezonder. Een actieve leefstijl is van groot belang voor een optimale groei en ontwikkeling van kinderen. De beperkingen als gevolg van het coronavirus maken dit extra zichtbaar. Het professionele netwerk ‘Zicht op Buiten’ bracht de gevolgen van te weinig buiten zijn en beweging voor de gezondheid van kinderen en jongeren in kaart, op het gebied van visus, motoriek, houding, overgewicht, slaap en psychosociale gezondheid. We bevelen sterk aan om 2 uur per dag naar buiten te gaan voor gezonde kinderogen, waarvan ten minste 1 uur matig-intensief bewegen om uithoudingsvermogen, spieren en botten te versterken. Andere leefstijlmaatregelen betreffen het verminderen van sedentair gedrag en regels over schermgebruik, evenals regelmatig afwisselen van zittende activiteiten.

## Abstract (en)

Outdoor play makes children healthier. An active lifestyle is particularly important for optimal growth and development of children. Restrictions due to the Corona virus make this more apparent. The professional network ‘View Outside’ collected the lifestyle consequences for visual, motoric, postural, weight, sleep and psychosocial youth health. We strongly recommend spending two hours a day outdoors, of which a minimum of one hour should be at least moderately intensive exercise. Other lifestyle measures are reducing sedentary behavior, rules on screen use and regular change of activities when sedentary.

## Buiten spelen

Buiten spelen hangt sterk samen met lichamelijk actief zijn en is daarom een belangrijke factor voor een gezonde ontwikkeling van kinderen [1]. Wereldwijd spelen kinderen steeds minder buiten en in Nederland blijkt uit een onderzoek van Jantje Beton uit 2018 dat 53% van de kinderen in hun vrije tijd vaker binnen spelen dan buiten [1, 2]. Er was de laatste vijf jaar een toename van het aantal kinderen dat minder buiten speelt dan dat zij zouden willen (19% versus 28% qua behoefte). Drie- en zesjarige kinderen uit het Rotterdamse onderzoek Generation R zijn gemiddeld 1,5 uur per dag buiten, op negenjarige leeftijd is dat gedaald naar nog maar 1 uur per dag.

Het professionele netwerk ‘Zicht op Buiten’ houdt zich al langer bezig met leefstijlverandering bij de jeugd, specifiek gericht op de toename van beeldschermgebruik [3]. Dit netwerk bracht de gevolgen van te weinig beweging en buiten zijn voor de gezondheid van kinderen en jongeren in kaart, op het gebied van visus, motoriek, houding, overgewicht, slaap en psychosociale gezondheid (zie tab. 1). Het netwerk bestaat uit onder anderen een oogarts-epidemioloog, orthoptist, jeugdartsen, kinderartsen, een jeugdverpleegkundige, orthopeed, bewegingswetenschapper-epidemioloog, gezondheidswetenschappers en psychologen. In november 2020 bood het netwerk een White Paper aan de secretaris-generaal van het ministerie van VWS aan om aandacht te vragen voor de gezondheidsgevolgen [4]. In dit artikel vatten we de gevolgen en praktische adviezen samen. We vragen tevens alle maatschappelijk betrokkenen om bij te dragen aan de oproep om kinderen gedurende 2 uur buiten te laten zijn en daarmee binnen zitten en overmatig schermgebruik te verminderen.

**Table 1:** 

Myopie (bijziendheid)	De helft van alle jongvolwassenen heeft myopie. Kinderen die veel dichtbij kijken (schermgebruik, lezen en dergelijke) én weinig buiten zijn, hebben een veel hoger risico om myopie te ontwikkelen dan kinderen die veel dichtbij kijken maar ook voldoende buiten spelen [4, 5]
Overgewicht	Dertien procent van de kinderen heeft overgewicht, van wie 2% ernstig (obesitas). Regelmatige lichamelijke activiteit van ten minste matige intensiteit, zoals buiten spelen, verlaagt de body mass index en vetmassa bij kinderen met overgewicht en obesitas [6]
Motorische ontwikkeling	De motorische fitheid van kinderen is de afgelopen decennia afgenomen. Kinderen die meer tijd binnen zitten en besteden aan tv-kijken hebben over het algemeen een slechtere aerobe fitheid [4, 6]
Houding, rug- en nekklachten	Lagerugklachten komen voor bij 34 tot 46% van de kinderen en jongeren, en dit lijkt toegenomen. Extreem veel en verkeerd zitten en gebogen op schermpjes turen gaat gepaard met ongelijkmatige belasting van de wervelkolom, wat leidt tot houdingsverval [7]
Slaapproblemen	Slaapproblemen komen voor bij 20 tot 30% van de peuters en kleuters, en bij 7 tot 36% van de adolescenten. Recente onderzoeken hebben laten zien dat kinderen die overdag meer buiten zijn, beter en langer slapen [8]
Psychosociale problemen	De laatste decennia zien we een toename van kinderen, adolescenten en jongvolwassenen met angststoornissen, depressieve gevoelens, suïcidale gedachten, hulpeloosheid en narcisme [9]. Er is steeds meer bewijs dat beweging niet alleen goed is voor de lichamelijke gezondheid, maar ook voor de mentale gezondheid [10]

## Leefstijladviezen

‘Voorkomen is beter dan genezen’, dus meer bewegen, zoals meer buiten spelen, maar ook meer bewegend leren op school, heeft een positief effect op de genoemde gevolgen. In Nederland heeft de Gezondheidsraad beweegrichtlijnen opgesteld voor volwassenen en kinderen. Die voor de laatsten komt overeen met de richtlijnen van de Wereldgezondheidsorganisatie WHO voor kinderen van vijf tot zeventien jaar:

1. minimaal 60 minuten per dag matig tot zwaar lichamelijke activiteit per dag;
2. meer beweging dan 60 minuten per dag levert extra gezondheidsvoordelen op;
3. minimaal drie keer per week spier- en botversterkende activiteiten.

De Jeugdgezondheidszorg (JGZ) Richtlijn Houding en beweging adviseert een gezonde afwisseling van zittende activiteiten. Dat versterkt de nek- en rugspieren. Ook wordt geadviseerd om elke dag buiten te spelen, ook als het regent, en om het beweegadvies van de Gezondheidsraad te volgen. De myopierichtlijn geeft het advies voor kinderen de 20-20-2-regel te volgen: na 20 minuten dichtbij kijken, 20 seconden in de verte kijken, plus 2 uur per dag naar buiten [5]. Uit een peiling van het Oogfonds blijkt dat kinderen deze regel duidelijk vinden en makkelijk onthouden [4]. De JGZ-richtlijn Gezonde slaap adviseert om kinderen iedere dag buiten te laten spelen, zodat zij voldoende bewegen en daglicht zien. Hierdoor vallen kinderen ’s avonds sneller in slaap en slapen ze dieper. Ook het spelelement in buiten spelen draagt bij aan een gezonde ontwikkeling. Kinderen die vaker buiten spelen hebben daardoor betere sociale vaardigheden. Voor adolescenten adviseert de richtlijn om elke dag naar buiten te gaan, omdat blootstelling aan zon- of daglicht helpt de biologische interne klok in de pas te houden. Daarnaast luidt het advies om regelmatig te bewegen. Dit kan zelfs ingezet worden als behandeling bij depressie – regelmatig joggen heeft een positief effect op adolescenten met depressieve symptomen [10].

Samenvattend, weinig buiten spelen, overmatig dichtbij kijken en langdurig beeldschermgebruik bij kinderen leveren op de korte en lange termijn gezondheidsproblemen op. De werkingsmechanismen achter de negatieve gezondheidseffecten op de ogen, het gewicht, de motoriek, het bewegingsapparaat, slaapkwaliteit en psychosociale gezondheid worden steeds duidelijker. Dat brengt ons tot de conclusie dat de verandering in leefstijl zich vertaalt in een toename van aandoeningen die later uitmonden in chronische ziektelast. Dit zal ook meer ziektekosten met zich meebrengen.

Wij vrezen dat deze gezondheidseffecten door de grote schaal waarop ze zich voordoen ook gevolgen zullen hebben voor de kansen van opgroeiende jongeren om duurzaam in de maatschappij te participeren in onderwijs en sport, en op de arbeidsmarkt. Als groep professionals willen wij onze zorgen uitspreken over de veranderende leefstijl en pleiten wij voor bewustwording bij iedereen die zich bezighoudt met de doelgroep jeugd [3].

## Vertaling naar de praktijk en kansen

Duurzame verandering kan alleen wanneer we als volwassenen het goede voorbeeld geven én onze sociale en fysieke omgeving veranderen. Professionals in de gezondheidszorg kunnen individueel adviezen geven, maar voor maatschappelijke impact is veel meer nodig. We willen de focus leggen op positieve gezondheid en, zeker als het om kinderen gaat, gezond gedrag benadrukken, zoals buiten zijn, buiten spelen, bewegen en veel afwisseling bij beeldschermgebruik.

De meest favoriete plaatsen van kinderen om buiten te zijn, zijn het schoolplein, de tuin en natuur of bos [2]. Een veilige speelomgeving is hierbij een voorwaarde – gemeenten kunnen hierin een rol spelen. Integraal beleid waarbij er samenwerking is tussen onderwijs, zorg, gemeenten en de landelijke overheid werkt, zoals blijkt uit de JOGG-aanpak en de Gezonde School-aanpak. De gemeente Venray heeft een leefstijlakkoord afgesloten waarbij er bijzondere verbindingen zijn gelegd tussen sport en andere domeinen, met het doel de leefstijl van de jeugd preventief te verbeteren. Een gezonde leefomgeving wordt ook gestimuleerd door andere gemeenten, zoals de regio IJsselland en zestien Zuid-Limburgse gemeenten. Daarnaast zijn er de afgelopen jaren geregeld regionale campagnes gevoerd om beweging en buiten spelen te stimuleren, zoals ‘Gratis bewegen, gewoon doen’ van de gemeente Groningen.

Verschillende organisaties en sectoren proberen op hun eigen gebied bij te dragen aan een gezonde en activerende omgeving voor jeugdigen. Neem het Kenniscentrum Sport en Bewegen en het Nederlands Centrum Jeugdgezondheid, die samen bewegingsvaardigheden bij jonge kinderen van nul tot vier jaar gaan stimuleren op consultatiebureaus. Ook het onderwijs besteedt steeds meer aandacht aan bewegend leren.

## Oproep

Toch vragen we ons af of de groeiende problemen die wij hebben geschetst bij iedereen goed op het netvlies staan en meer nog, of voldoende mensen en organisaties zich richten op preventie en oplossingen met een duurzame impact. Het doet ons ons deugd dat er in de *Landelijke nota gezondheidsbeleid 2020-2024 – Gezondheid breed op de agenda* aandacht is voor de fysieke en sociale omgeving. Hiermee wordt de prioritering in de publieke gezondheid door het rijk aangegeven. De vertaling naar de praktijk via de lokale nota’s jeugdbeleid is nu volop in gang en er liggen niet alleen kansen om de leefomgeving te veranderen, maar ook om gedrag te beïnvloeden. Er zijn wetenschappelijk erkende interventies beschikbaar (zie de Interventiedatabase van het RIVM Loket Gezond Leven), maar er is veel meer nodig.

Het netwerk ‘Zicht op Buiten’ is bezig om maatschappelijke bewustwording te creëren door tijdens de Buitenspeelweek van 2021 een webinar te organiseren voor alle beleidsadviseurs. In het najaar volgt een webinar voor ouders en onderwijs. Maar het netwerk heeft hulp nodig bij de volgende stappen. Wat kunnen we nog meer doen om gezond gedrag (2 uur per dag buiten zijn en gezond beeldschermgebruik) te promoten?

Wij, het netwerk ‘Zicht op Buiten’, doen daarom een oproep aan alle betrokkenen, ouders en andere opvoeders, individuele medewerkers en organisaties in het onderwijs, gezondheidszorg, sport, werkgevers en werknemers, gemeenten en andere overheden om mee te denken over de volgende te nemen stappen.
